# The Role of Cancer-Associated Fibroblasts in Ovarian Cancer

**DOI:** 10.3390/cancers14112637

**Published:** 2022-05-26

**Authors:** Mo Zhang, Zhixian Chen, Yan Wang, Hongbo Zhao, Yan Du

**Affiliations:** 1 Clinical Research Unit, Obstetrics and Gynecology Hospital of Fudan University, Shanghai 200011, China; zhangmodoct@163.com (M.Z.); chenzx1997@163.com (Z.C.); wangyan19820115@163.com (Y.W.); 2 Department of Obstetrics and Gynecology, Shanghai Medical School, Fudan University, Shanghai 200032, China; 3 Shanghai Key Laboratory of Female Reproductive Endocrine Related Diseases, Shanghai 200011, China

**Keywords:** cancer-associated fibroblasts, ovarian cancer, tumor microenvironment, stroma

## Abstract

**Simple Summary:**

Ovarian cancer is a lethal gynecologic tumor and is generally resistant to conventional treatments. Stable cancer-associated fibroblasts (CAFs) are important cellular components in the ovarian cancer tumor microenvironment and may provide novel resources for future treatment strategies. Different subtypes of CAFs display specific functions in tumor pathogenesis and various CAF markers suggest potential treatment targets. Several clinical or preclinical trials have targeted stromal fibroblasts and focused on the properties of CAFs to enhance ovarian cancer treatment efficacy. This review concentrates on the origins, subtypes, and activation of CAFs, as well as specific roles of CAFs in regulating tumor development and drug resistance, and aims to provide potential and prospective targets for improving the therapeutic efficacy of ovarian cancer treatment.

**Abstract:**

Ovarian cancer is a lethal gynecologic tumor and is generally resistant to conventional treatments. Stable cancer-associated fibroblasts (CAFs) are important cellular components in the ovarian cancer tumor microenvironment and may provide novel resources for future treatment strategies. Different subtypes of CAFs display specific functions in tumor pathogenesis and various CAF markers suggest potential treatment targets, such as FAP and GPR77. Both autocrine and paracrine cytokines play important roles in the CAF activation process and regulate tumor progression. Downstream mediators and pathways, including IL-6, TGF-β, NF-κB, mitogen-activated protein kinase (MAPK), and AKT/mTOR/(p70S6K), play important roles in the initiation, proliferation, invasiveness, and metastasis of ovarian cancer cells and also participate in angiogenesis, therapeutic resistance, and other biological processes. Several clinical or preclinical trials have targeted stromal fibroblasts and focused on the properties of CAFs to enhance ovarian cancer treatment outcomes. This review concentrates on the origins, subtypes, and activation of CAFs, as well as specific roles of CAFs in regulating tumor development and drug resistance, and aims to provide potential and prospective targets for improving the therapeutic efficacy of ovarian cancer treatment.

## 1. Introduction

Globally, there are about 300,000 new cases of and 185,000 deaths due to ovarian cancer, making it the third most common gynecologic cancer and the second leading cause of reproductive cancer death among women [1,2]. In 2015, there were about 52,100 new cases of and 22,500 deaths caused by ovarian cancer in China [3], causing significant health issues and seriously threatening women’s health. Due to various pathological types, limited screening methods, and lack of distinct clinical manifestations, 75% of patients are diagnosed at an advanced stage and the prognosis is not optimistic [4]. At present, the mainstay treatment of ovarian cancer is surgical cytoreduction to R0, followed by carboplatin and paclitaxel combined chemotherapy [5]. Further maintenance therapies may be applied after first-line chemotherapy, which include poly ADP-ribose polymerase (PARP) inhibitors, anti-angiogenesis agents or monoclonal antibodies [6]. Although more than 80% of patients are initially sensitive to treatment, the majority of them go on to develop chemotherapy resistance, resulting in advanced recurrence and eventually death [7]. It is reported that the overall 5-year survival rate of advanced patients is approximately 30%, and prognoses are not optimistic even in high-resource countries such as the United States, where the rate is only about 47% [1]. Therefore, there is an urgent need to better understand the heterogeneity and biology of ovarian cancer so as to develop or refine treatment strategies and improve quality of life.

Since Paget first proposed the ‘Seed and Soil’ theory in 1889 [8], it has been gradually recognized that apart from the cancer cells, stable stromal cells with few mutations in the tumor microenvironment (TME) are also of great importance [9]. The interplay between tumor and stromal cells contributes greatly to tumorigenesis, progression, and therapeutic resistance [10]. Some of the most significant stromal components are cancer-associated fibroblasts (CAFs), which modulate cancer progression and treatment responses in many cancer types [10,11]. CAFs can form robust crosstalk with cancer cells, participate in various biological processes, including wound healing and inflammatory processes, tumor initiation, progression, and immune exclusion, and may also contribute to therapeutic failure, especially chemoresistance [10,11,12]. Therefore, CAFs are considered to be notable tumor-promoting players. Treatment barriers in ovarian cancer have been increasingly ascribed to angiogenesis, stromal reprogramming, extracellular matrix remodeling (ECM), and drug delivery [13,14]. In principle, a better understanding of the intricate mechanisms of CAFs in tumor pathophysiology could pave the way for CAF-targeted therapeutics. Interestingly, several studies focusing on Shh-deficient signaling indicated that, besides supporting tumor pathogenesis, CAFs also had tumor-restraining functions [15,16]. A population-based study has demonstrated improved 10-year survival in breast cancer patients receiving breast-conserving surgery plus radiotherapy compared with mastectomy, which may partially be explained by remnant tumor stroma after surgery. The stromal components could induce long-lasting antitumor immunity and restrain tumor progression, and therefore may improve clinical outcomes [17,18]. The available evidence has suggested that in-depth studies of CAFs may aid in elucidating the mechanisms of ovarian cancer progression, dissemination, and therapeutic resistance. This review aims to update the current understanding of tumor-associated fibroblasts in ovarian cancer and potential therapeutic strategies targeting the stromal ‘soil’.

## 2. Origins, Classifications, and Activation Mechanisms of CAFs

**Figure 1:**
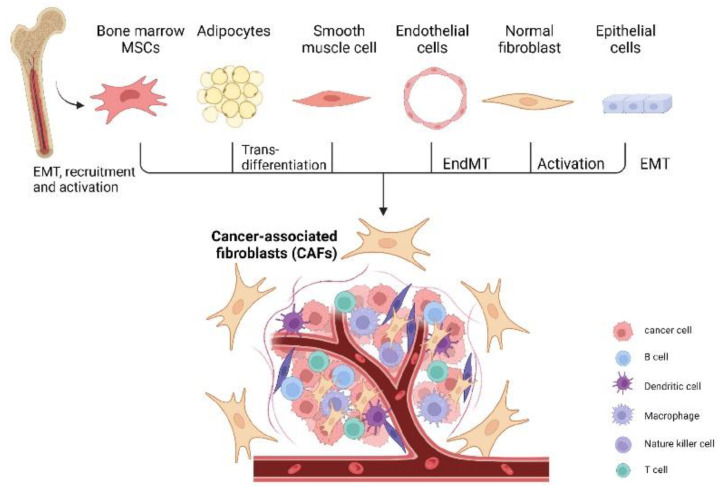
Origins of ovarian cancer-associated fibroblasts. CAFs are important components in the tumor microenvironment and may potentially derive from several cellular sources, including normal fibroblasts (which can convert into CAFs via activation), epithelial cells (through EMT), smooth muscle cells (through trans-differentiation), adipocytes (through trans-differentiation), endothelial cells (EndMT), and bone marrow MSCs (through EMT, recruitment, and activation), among other cells. Image created with BioRender.com (accessed on 19 April 2022). CAFs: cancer-associated fibroblasts; EMT: epithelial-to-mesenchymal transition; EndMT: endothelial-to-mesenchymal transition; MSCs: mesenchymal stem cells.

**Figure 2:**
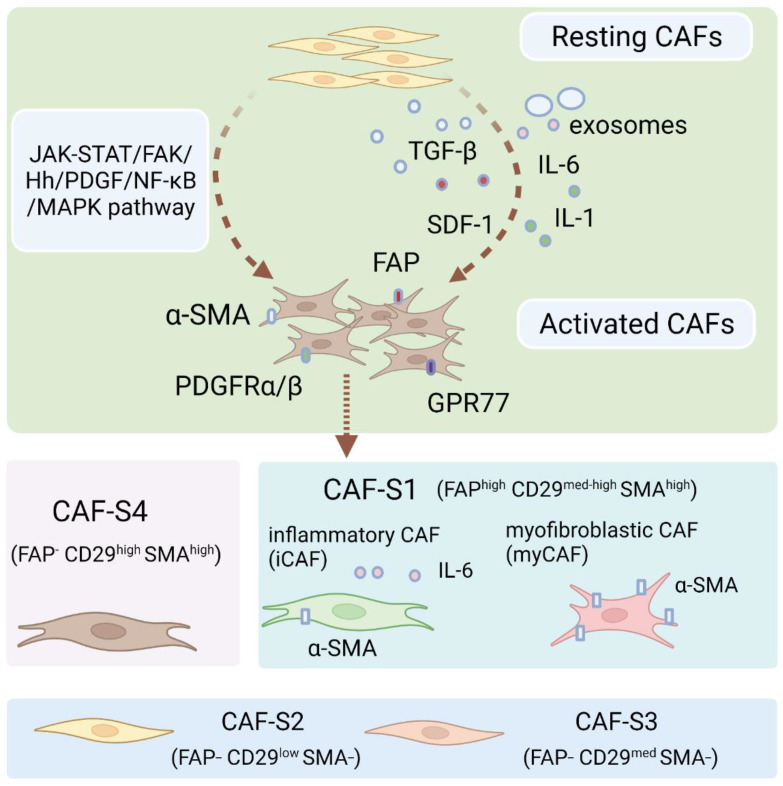
Pathways and mediators associated with CAFs activation. Quiescent fibroblasts can be transformed into an activated state through inflammatory cytokines and several pathways. Based on the different expressions of surface markers, including α-SMA, FAP, CD29, PDGFRβ, FSP1, and CAV1, CAFs are classified into four subsets in some cancers. Single-cell sequencing analysis of CAF-S1 in ovarian cancer has identified two subsets, iCAFs and myCAFs. Image created with BioRender.com (accessed on 25 May 2022).

CAFs can be broadly defined as fibroblasts displaying complex heterogeneity. CAFs usually locate in or near a tumor and are crucial components of the tumor microenvironment [19]. CAFs may be derived from normal fibroblasts, bone marrow mesenchymal stem cells (MSCs), epithelial cells, endothelial cells, or adipocytes (Figure 1) [20,21,22,23,24,25]. CAFs exist in two primary cellular states, namely, quiescent and activated. Quiescent CAFs, also known as resting CAFs, are often characterized by Vimentin (VIM). Quiescent CAFs mainly represent normal fibroblasts and usually have lower proliferative and metabolic capacities than activated CAFs [19]. Activated CAFs express various markers, including α-smooth muscle actin (α-SMA), fibroblast activation protein (FAP), low caveolin1 (CAV1), CD10, integrin β1 (CD29), platelet-derived growth factor receptor-α/-β (PDGFRα/β), and G protein-coupled receptor 77 (GPR77) [8,11,26,27,28,29,30]. Resting CAFs can be transformed into the active state via different pathways, including the JAK/STAT signaling pathway, the focal adhesion kinase (FAK) pathway [31], the Hedgehog signaling pathway [32], and the platelet-derived growth factor signaling (PDGF) pathway (Figure 2) [33]. Some cytokines, including IL-1, IL-6, NF-κB, and TGF-β, also play pivotal roles in the activation process [33,34,35,36]. Autocrine signaling loops, which are mediated by TGF-β and SDF-1, contribute to the tumor-promoting CAF phenotype [37]. In addition, exosomes released by stromal cells can deliver microRNAs to activate CAFs at a post-transcriptional level, especially through the downregulation of miRNA-31/-214 and upregulation of miRNA-155 [38,39]. Remodeling of ECM can also activate CAFs [40]. In ovarian cancer, four subtypes of CAF populations (CAF-S1 to CAF-S4) have been discovered by analyzing CAF markers of FAP, α-SMA, CD29, PDGFRβ, FSP1, and caveolin 1 (CAV1). Interestingly, CAF-S2 (FAP^−^ CD29^low^ SMA^−^) and CAF-S3 (FAP^−^ CD29^med^ SMA^−^) resemble normal fibroblasts, while CAF-S1 (FAP^high^ CD29^med-high^ SMA^high^) and CAF-S4 (FAP^−^ CD29^high^ SMA^high^) are restricted in cancer and metastatic sites, resembling the activated state [41]. CAF subtypes may be correlated with ovarian cancer subtypes and affect clinical manifestations and outcomes. Especially in the mesenchymal high-grade serous ovarian cancer (HGSOC) subtype, high concentrations of CAF-S1 fibroblasts are associated with poor patient survival [19,41]. CAF-S1 facilitates the immunosuppressive activities of tumors by enhancing recruitment, differentiation, and activation of CD25^+^FOXP3^+^ T cells [41]. The existence of four subtypes of CAFs has also been confirmed in pancreatic cancer [42]. Furthermore, single-cell sequencing analysis of ovarian cancer has confirmed the existence of distinct subsets of CAF-S1, including myofibroblasts CAFs (myCAFs) and inflammatory CAFs (iCAFs). myCAFs, with high expression of α-SMA, are located adjacent to cancer cells and produce dense matrices to support tumorigenesis, while iCAFs, with low α-SMA expression, are distal to tumor edges and characterized by secreting inflammatory cytokines, such as IL-6, and consequently induce an immunosuppressive microenvironment [19,41,42,43]. Single-cell sequencing analysis of CAF-S1 collected from malignant ascites in ovarian cancer has identified distinct subpopulations of inflammatory CAFs expressing immunoregulatory programs, such as complement factors, cytokines, and chemokines. iCAFs secrete IL-6 and other cytokines and share activation of the JAK/STAT pathway with tumor cells to cause ascites, promote tumor growth, and subsequently promote therapeutic failure [44]. In conclusion, phenotypes, features, and heterogeneity are defined and characterized by single-cell analysis of ovarian cancer. More specific classifications of CAF subtypes may help to better elucidate ovarian cancer pathogenic processes and drug resistance.

## 3. Mechanisms of CAFs in the Progression of Ovarian Cancer 

**Figure 3:**
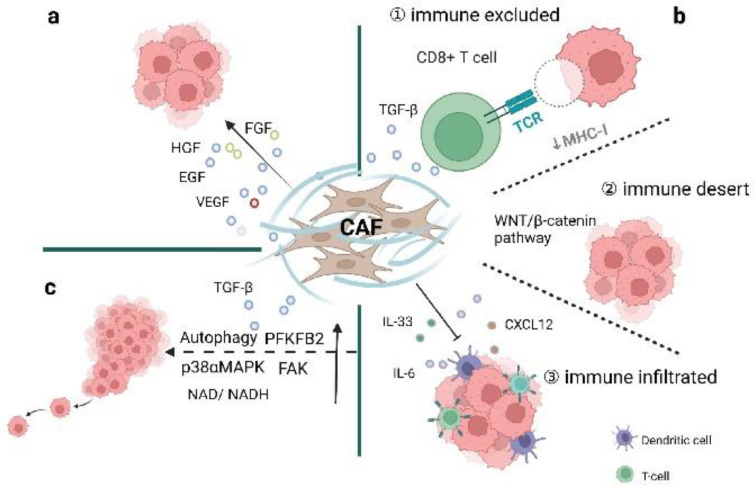
Mechanisms of CAFs in ovarian cancer progression and dissemination. CAFs can regulate the growth, proliferation, and metastasis of ovarian cancer in different ways, including (**a**) secreting cytokines, (**b**) shaping the immune microenvironment, and (**c**) establishing metabolic crosstalk. Image created with BioRender.com (accessed on 24 April 2022).

Compared to normal fibroblasts, CAFs have faster proliferative capacities and are characterized by higher metabolic states. Several neoplastic signaling pathways, such as the NF-κB signaling pathway, are abnormally activated in CAFs. In this section, we discuss the biological behaviors of CAFs and how they function to modulate tumor progression. CAFs can directly act on adjacent tumor cells through paracrine modes and can also indirectly regulate immunity and affect tumor metabolism (Figure 3) [11]. Detailed mediators and mechanisms are summarized in Table 1.

### 3.1. CAF-Secreted Cytokines Facilitate Tumor Growth, Proliferation, and Metastasis

Tumor fibroblasts can release a variety of cytokines to act on different signaling pathways in the tumor microenvironment via paracrine modes, thus directly stimulating the proliferation, differentiation, invasion, and metastasis of surrounding tumor cells. 

TGF-β released from myofibroblasts is a vital mediator in regulating cancer–stroma interplay to induce tumor initiation and immune escape [51]. *HOXA9*, a Müllerian-patterning gene, is highly expressed in ovarian cancer SKOV3 cells. The patterning gene can activate the transcription of the *TGF-β2* encoding gene. Then, the activated TGF-β2 may be able to induce CAF expression of CXCL12, IL-6, and VEGF-A to form a favorable microenvironment for EOC progression (Figure 3a) [63]. For example, many TGF-β signaling-related genes derived from CAFs play important roles in the crosstalk between fibroblasts and ovarian cancer, including periostin (*POSTN*), collagen type XI alpha 1 (*COL11A1*), collagen triple helix repeat containing-1 (*CTHRC1*), and versican (*VCAN*) [45,46,47,48,64,65,66]. These proteins exert effects through the TGF-β signaling pathway to participate in CAFs activation and tumor pathogenic processes. Upregulated CTHRC1 enhances tumor invasion and migration via EGFR/ERK1/2/AKT signaling. It also participates in the immune response and angiogenesis to stimulate tumor progression [45]. The upregulated COL11A1 serves as a mediator of stroma–cancer cell crosstalk and plays important roles in activating CAFs by regulating TGF-β3 through the NF-κB/IGFBP2 axis [46]. The processes of promoting tumor formation and activating CAFs can be attuned by TGF-β3 antibodies, suggesting a promising treatment target in COL11A1-positive ovarian cancer [46]. COL11A1 is also associated with tumor aggressiveness and poor clinical outcomes via the TGF/β1/MMP3 axis [47]. CAFs-derived POSTN activates fibroblasts through TGF-β1 and exerts effects on the migration and invasiveness of ovarian cancer via activating the PI3K/Akt pathway. Pro-metastatic properties and remodeling of the pro-metastatic microenvironment partly rely on stromal-derived POSTN protein [65]. VCAN is secreted by CAFs and its expression level is regulated through TGF-β receptor type II and SMAD signaling. Upregulation of VCAN protein may promote cell motility and the invasiveness of advanced ovarian cancer through activation of the NF-κB signaling pathway and elevated expression of CD44, matrix metalloproteinase-9 (MMP-9), and the hyaluronan-mediated motility receptor [48].

CAFs-derived hepatocyte growth factor (HGF) or human recombinant HGF can promote cell proliferation in the ovarian cancer cell lines SKOV3 and HO-8910 by activating the c-Met/PI3K/Akt and GRP78 signaling pathways. HGF can also weaken paclitaxel efficacy to induce chemotherapeutic failure. The above effects can be blocked by anti-HGF and c-Met inhibitors INCB28060 [49]. As for CAF-related gene fibroblast growth factor-1 (*FGF-1*), underlying mechanisms regulating tumor progression include phosphorylation of FGF-4, activation of the mitogen-activated protein kinase/extracellular signal-regulated protein kinase (MAPK/ERK) pathway, and also upregulated expression of EMT-related genes, such as *Snail-1* and *MMP3* [50]. The FGF-1/FGF-4 axis may provide a novel strategy for ovarian cancer treatment. In addition, ECM remodeling is also considered to be an inducer that promotes the invasion and metastasis of SKOV3 ovarian cancer cells [50]. In high-grade serous ovarian cancer (HGSOC), integrin α5^high^ (ITGA5^high^) ascitic tumor cells (ATCs) are recruited by CAFs to form heterotypic spheroids. The aggregates are also termed metastatic units (MUs). ITGA5 is indispensable in the formation of MUs. CAFs-secreted epidermal growth factor (EGF) could sustain ITGA5 expression and maintain the aggregates formed by CAFs and ACTs. CAFs-centered MUs could promote early peritoneal dissemination of HGSOC and then accelerate the development of ascites [67]. iCAFs share the activation of the JAK/STAT pathway with tumor cells to shape the ascites system and promote tumor growth and induce therapeutic resistance [44]. Studies have indicated that Hedgehog (Hh) signaling could contribute to the modulation of the stromal microenvironment and generate a favorable niche for cancer metastasis. It has been shown that Sonic Hedgehog (SHH) could induce VEGF-C expression and activate Hh signaling in CAFs via a paracrine mode to accelerate tumorigenesis and lymphangiogenesis in ovarian cancer. The Hh/VEGF-C signaling axis could also be a promising treatment target for ovarian cancer [68]. With the increased understanding of CAFs, it is believed that targeting CAFs-secreted cytokines can arrest ovarian cancer progression, thus providing a promising way for developing novel treatment strategies.

### 3.2. Immunoregulatory Roles of CAFs in Shaping the Tumor Microenvironment

The immune system may recognize and attack ovarian cancer components. Lymphocytes, especially CD8^+^ T cells [69], can infiltrate carcinomas [70], identify tumor antigens, exhibit oligoclonal expansion, and yield cytotoxic responses to tumor tissues [71]. Several studies have also demonstrated the link between tumor-infiltrating T cells and improved clinical outcomes in ovarian cancer patients [71,72,73,74,75]. Meanwhile, interactions between CAFs and immune components can regulate the tumor immune microenvironment (TIME) to inhibit anti-tumor activities. CAFs can recruit immune cells to participate in tumor progression through the secretion of chemokines and cytokines and regulation of effective signaling pathways. To date, different approaches have been applied in classifying tumor-immune phenotypes to better characterize the immune presence and understand the different TIME conditions. The ICON7 phase Ⅲ clinical trial utilizing digital pathology with transcriptome analysis has described three tumor-immune phenotypes in which CAFs play a role, including inflamed, excluded, and desert subtypes [19,43,72]. The three tumor-immune phenotypes show specific cellular levels of T cells in cancers. CD8+ T cells mainly infiltrate the tumor epithelium in the immune-inflamed phenotype and accumulate in the tumor stroma in the immune-excluded phenotype. In the immune-desert phenotype, CD8+ T cells are either absent or present in very low numbers [76]. Apart from differences at the cellular levels, the related molecular mechanisms also vary (Figure 3b).

One study integrating digital pathology and transcriptome analysis has identified two crucial mechanisms in T cell-excluded tumors (Figure 3(b①)). One involves contributions to the loss of MHC-I presentation on tumor cells; the other relies on upregulated TGF-β playing important roles in ECM formation and the activation of stromal components to enhance T cell exclusion [72]. Unlike other types of cancers, genetic mutations in *MHC-I* genes are rare in ovarian cancer [77]. However, epigenetic regulation plays an important role in ovarian cancer. DNA methylation contributes to MHC-I loss in ovarian cancer and reduces tumor antigen expression and untimely leads to immune exclusion. Specifically, MHC-I loss can be reversed by treatment with DNA methyltransferase (DNMT) inhibitors, indicating that epigenetic regulation might be a promising therapeutic target [72]. TGF-β, produced by myofibroblasts as well as other cells in the TME [52], regulates innate and adaptive immunity via inhibitory effects on natural killer (NK) cells, CD4^+^/CD8^+^ T cells, macrophages, and other effector immune cells [53]. In addition, TGF-β also enhances the generation and function of regulatory T cells (Treg), Th17, Th9, and Tfh cells, to form a negative regulatory immune network and support tumor occurrence [51,53,54]. Interestingly, TGF-β could also reduce the expression of MHC-I in ovarian cancer, which could be restored to pre-treatment levels by the TGF-β inhibitor Galunisertib [72]. Cancer cells rely on the immunosuppressive role of TGF-β to evade immune surveillance and weaken immunotherapeutic efficacy [53]. Apart from low expression of IFN-γ and antigen presentation, T cell-excluded ovarian cancer also lacks chemokine signaling-related genes. The above two mediators (MHC-I and TGF-β) are considered as barriers to the distribution and function of cytotoxic T cells and are involved in cancer pathogenesis and closely associated with clinical outcomes.

Immune-desert ovarian cancer is characterized by the lowest interferon and inflammatory responses and non-T cell infiltration (Figure 3(b②)) [19,78]. In addition, the MYC and WNT/β-catenin signaling pathways are abundant in high tumor cellularity, which is closely associated with immune exclusion [55,79]. Furthermore, the WNT/β-catenin signaling pathway is also correlated with resistance to anti-PD-L1 and anti-CTLA4 combined therapy [80]. Studies have also explored the interaction of metabolism and tumor-immune subtypes [81]. It has been suggested that upregulation of hormone biosynthesis and low immune cell infiltration are the two main characteristics of the ‘immune-desert–endocrine subtype’, which is negatively correlated with MHC molecules and multiple immune cells, such as dendritic cells, and finally leads to the immune-desert type [81]. 

Accumulating evidence has shown that CAFs play important roles in regulating the immune activities of both innate and adaptive cells to suppress anti-tumor immunity. For example, a high number of iCAFs in the TME could suppress both innate and adaptive immune responses in the tumor microenvironment through their secretory profiles [19,29]. iCAFs may secrete IL-6, CXCL12, and CXL2 to retain immune-suppressive functions and negatively influence anti-tumor effects (Figure 3(b③)) [29]. Highly expressed presenilin 1 (PS1) in CAFs attenuates the proliferation and infiltration of CD8^+^ T cells and dendritic cells via the WNT/β-catenin pathway in ovarian cancer [55]. CAFs are the main cellular sources of IL-33 in breast cancer metastasis. Upregulation of IL-33 could drive type 2 immunity and recruit eosinophils, neutrophils, and monocytes to facilitate metastatic behavior [57]. CAFs also produce chemokines to attract immunosuppressive cells, such as Treg and myeloid-derived suppressor cells (MDSCs) [41,82]. Previous studies have shown that CXCL12 wraps tumor cells through binding to CXCR4 and thereby prevents effector T cells from contacting cancer cells and consequently leads to immune escape/immunosuppression activities [56]. The mesenchymal HGSOC subtype is enriched with CAF-S1 fibroblasts (FAP^high^ CD29^med-high^ SMA^high^), which are the main cellular sources of CXCL12. Studies have shown that miR200-regulated CXCL12β accumulates in CAF-S1 and plays a crucial role in immunosuppressive functions with the help of CD25^+^ FOXP3^+^ T cells (Treg) [41]. CXCR2 ligands, including CXCL1 and CXCL2, attract MDSC infiltration in Snail-expressing mice to promote ovarian cancer progression [82]. Metabolic stress may stimulate impaired antitumor immunity in immune-infiltrating tumors [81]. iCAF subsets also produce certain cytokines, especially IL-6, which presents a pro-tumor function and is closely associated with chemoresistance and poor prognosis [83]. Apart from this, ovarian cancer-associated exosomes can also produce IL-6 from monocytes and activate the STAT3 pathway to protect cancer cells from immune attack [58]. On the other hand, IL-6 could present an anti-tumor function by inducing the transformation of CD8^+^ T cells into highly cytotoxic cells [84]. In a mouse model of pancreatic ductal adenocarcinoma (PDAC), upregulation of CD4^+^Foxp3^+^ Treg has been observed in myofibroblast-depleted mouse tumors. Tregs-suppressed immune surveillance has led to enhanced tumor progression and invasion and also correlated with reduced survival. These results suggest that myofibroblasts play a protective role in the host [15]. At the same time, fewer myofibroblasts are correlated with reduced survival in PDAC patients [15]. Therefore, we speculate that CAFs may also exert an inhibitory effect on tumor cell growth in ovarian cancer. CAFs play crucial roles in differentiating spatial distributions of T cells in the tumor microenvironment. The three tumor-immune phenotypes in which CAFs play a role have provided an important framework for better understanding TME conditions which will help guide the development of immunotherapy.

### 3.3. Metabolic Processes Involved in Ovarian Cancer Pathogenesis

Recently, evidence has shown that CAFs participate in the metabolic processes of ovarian cancer through various modes, including cytokines, metabolites, TGF-β, and extracellular vesicles with metabolites [85]. During the early stage of tumor development when nutrients are insufficient for tumorigenesis, CAFs provide energy through the tricarboxylic acid cycle (TCA) to maintain tumor biological processes in what is known as the ‘reverse Warburg effect’ [59]. Recent studies have shown that cancer and stromal cells constitute a cellular ecosystem and create a shared metabolic environment. Tumor cells maintain tumorigenesis by establishing bidirectional communication with CAFs (Figure 3c) [86]. Autophagy plays an important role in the shared metabolic environment [86]. For example, in a co-culture system of ovarian cancer cells and fibroblasts, mapping of bidirectional signaling using quantitative phosphoproteomics revealed that activation of p38αMAPK kinase in CAFs and phosphoglucomutase 1 involved in glycogen metabolism can provide a tunnel for glycolysis and supply energy for tumor cell proliferation, invasion, and metastasis [59]. At the same time, decreased p38αMAPK and activation of glycogen phosphorylase can inhibit tumor metastasis [59]. Zhao et al. have reported that CAFs with high expression of CXCL14 could increase the expression of long non-coding RNA LINC00092, which consequently affects glycolysis by binding to the 6-phosphofructo-2-kinase/fructose-2,6-biphosphatase 2 (PFKFB2) glycolytic enzyme and promoting tumor metastasis with the support of surrounding CAFs [60]. It has been reported that low expression of focal adhesion kinase (FAK) in CAFs is associated with reduced overall survival in breast cancer and pancreatic cancer, possibly through the promotion of malignant cell glycolysis and growth via a paracrine mode independent of genetic mutations. CCR1/CCR2 on cancer cells and the activation of protein kinase A (PKA) play important roles in the increased production of chemokines [87]. Extracellular microRNAs can mediate metabolic reprogramming in stroma to support tumor progression under the shared metabolic environment [88]. Breast cancer-secreted exosomal miR-105, in particular, could enhance the metabolic programming of glucose and glutamine to fuel adjacent cancer cells via activation of MYC signaling in CAFs [88]. Given a lack of nutrients in the environment, CAFs may detoxify metabolic wastes into metabolites rich in energy. miR-105 could mediate metabolic reprogramming of stromal cells to regulate the shared metabolic environment and thus maintain tumor progression under different conditions [88]. It has been observed that lactate shuttled between tumor cells and stromal cells changed the NAD/NADH ratio in tumor cells, resulting in enhanced mitochondrial quality and activity in both in vivo and in vitro models of prostate cancer. On the other hand, tumor cells can hijack the functional mitochondrial complexes of CAFs by building cell bridges to ensure their own energy supply [89]. 

Autophagy, a catabolic process, may maintain cell metabolism and renewal of organelles to sustain homeostasis [90]. Higher levels of reactive oxygen (ROS) and activation of NF-κB can induce autophagy in CAFs, which, in turn, can protect CAFs from oxidative damage [61]. This catabolic process of producing metabolites can lead to metabolic reprogramming and drive the metabolic coupling of stroma and epithelium. Ovarian cancer cells can induce the upregulation of MCT4 and loss of expression of Cav-1 via autophagy, leading to a microenvironment favorable to tumor growth [62]. Interference with the metabolic pathway between ovarian tumor cells and fibroblasts could be a promising strategy to prevent the progression and recurrence of ovarian cancer. 

## 4. The Role of CAFs in the Therapeutic Resistance of Ovarian Cancer 

Chemotherapy plays an important role in the comprehensive treatment of ovarian cancer. Although more than 80% of patients respond well to treatment at an early stage, most patients develop drug resistance at the late stage and eventually suffer relapse and/or death [7]. Although mechanisms of drug resistance are still elusive, immune exclusion triggered by Treg or other immune components may play an important role [91,92]. The formation of extracellular matrices can interfere with drug transport and affect drug efficacy [92,93]. Some specific CAFs subtypes, such as CD10^+^ GPR77^+^ CAFs, or CAFs markers, such as CD44, may promote chemoresistance by sustaining cancer cell stemness [94,95]. Other mechanisms may be related to metabolic activities, such as autophagy and inhibition of cancer cell apoptosis [96,97,98]. Single-cell RNA sequencing (scRNA-seq) has been applied to analyze fibroblast heterogeneity. Results have shown that CAF-S1, which is characterized by TGF-β signaling, is correlated with immunosuppression and indicates primary resistance to chemotherapy. CAF-S1 upregulates the protein levels of PD-1 and CTLA-4 in regulatory T lymphocytes (Treg), which, in turn, increases levels of CAF-S1. On the other hand, upregulated levels of PD-1 and CTLA-4 proteins may serve as promising checkpoints which may pave the way for combining immunotherapy with targeted therapies [91]. Many chemotherapeutic agents must pass through the blood vessels and ECM to reach a tumor and exert effects. During this process, CAFs can obstruct the transportation of chemotherapeutic agents and impair chemotherapeutic efficacy by creating physical barriers and microvascular compression. For example, cysteine and glutathione produced by CAFs can inhibit the accumulation of cisplatin in the nucleus of ovarian cancer, resulting in platinum resistance [92]. Effector CD8^+^ T lymphocytes could affect the metabolic process of cysteine and glutathione in fibroblasts, creating chemoresistance via the JAK/STAT1 pathway or lowering medication efficacy by modifying cysteine and glutathione metabolism [92]. NF-κB continuously activates CD10^+^ GPR77^+^ CAFs through p56 phosphorylation and acetylation. Maintained by complementary Ca5 and GPR77 receptor signaling, CD10^+^ GPR77^+^ CAF activation provides a supportive environment for tumor stem cells and chemoresistance [94]. It has also been observed that CAFs derived from wild-type mice express high levels of CD44 in hypoxic and low-nutrition environments, which could maintain the stemness of cancer stem cells, while CD44-deficient CAFs do not have these properties [95]. Another study of CAFs and SKOV3 ovarian tumor cells has shown that increased level of ROS could promote autophagy of tumor cells and enhance the expression of drug resistance-related genes, such as *YAP, CTGF*, and *Cyr61* [96]. Functional studies have also confirmed that microRNA-21 (miR21) in exosomes transferred from neighboring CAFs could inhibit ovarian cancer apoptosis and generate chemotherapy failure [97], which has provided an alternative strategy for suppressing tumorigenesis and treatment resistance. In addition, CAFs could also affect ovarian cancer chemotherapy resistance by directly acting on XIAP and regulating the PI3K/AKT signaling pathway [98]. Many chemotherapeutic agents must be degraded into active forms to exert their antitumor effects. For example, gemcitabine, a pyrimidine antagonist, should be metabolized intracellularly to active metabolites to inhibit DNA synthesis. Gemcitabine is used in the treatment of ovarian cancer, as a single agent or together with carboplatin and/or bevacizumab in recurrent ovarian cancer, with survival benefits reported in clinical trials [99,100]. However, studies directly investigating the effects of gemcitabine on CAFs have mostly examined pancreatic cancers. TGF-β-induced stromal CYR61 negatively regulates the expression of the nucleoside transporters Hent1 and Hcnt3 in pancreatic tumor cells and significantly reduces the uptake of gemcitabine by cells, which is linked to gemcitabine resistance [93]. Although radiotherapy is not a standard treatment for ovarian cancer, it is also used when single recurrences occur after first-line treatment [101]. Similar to chemotherapy, CAFs have been proven to regulate the radiation response of malignant tumors via a paracrine mode or direct interactions during tumor radiotherapy [102]. Chemotherapy, radiotherapy, and immunotherapy all induce DNA damage in tumor cells, so survival and apoptosis-related signals can also affect the resistance to radiation-induced DNA damage, such as CAFs stimulation of MAPK, the AKT signaling pathway, and the β1-integrin-FAK signaling pathway [103]. It has been reported that TGF-β accelerates EMT progression and causes E-cadherin loss, and therefore induces radiotherapy tolerance in pancreatic cancer [104,105]. Additionally, CAFs affect the reactivity of tumor cells to checkpoint inhibitors by forming immunosuppressive tumor microenvironments. For example, in pancreatic ductal carcinoma, CXCL12 from FAP^+^ CAFs can wrap tumor cells and inhibit the accumulation of T cells in tumor sites. Depletion of FAP^+^ CAFs enables immunological checkpoint antagonists, such as α-CTLA-4 and α-PD-L1, to exert antitumor effects. CXCL-12 receptor 4 (CXCR4) inhibitors can be applied to recruit T cells to accumulate in cancer cells as well as cooperate with α-PD-L1 to significantly reduce the number of tumor cells [56]. What is more, high-dose radiotherapy can reprogram cancer immunity to activate both innate and adaptive immunity by upregulating the expression of MHC class I molecules and death receptors on tumor cells [106].

## 5. Therapeutic Prospective of CAFs in Ovarian Cancer 

**Figure 4:**
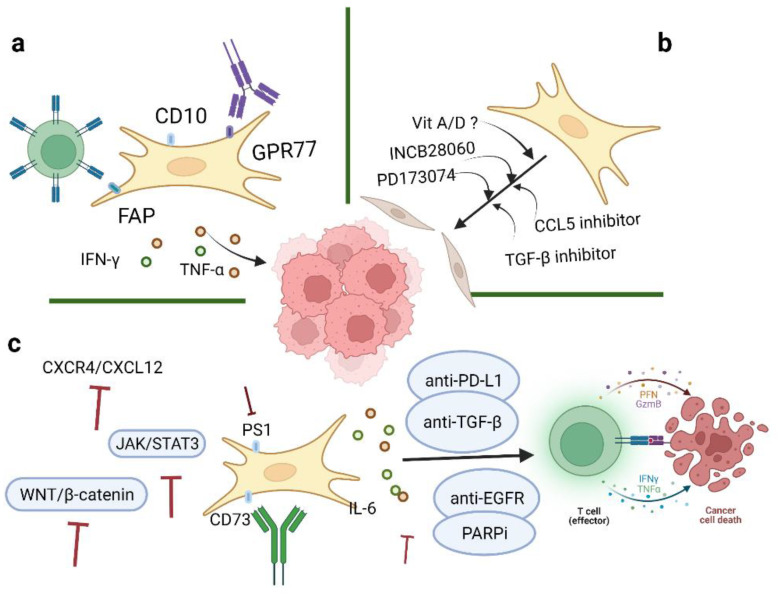
Therapeutic strategies of CAFs in ovarian cancer. Three aspects of strategies targeting CAFs in TME include: (**a**) Depletion of CAFs via specific surface markers; (**b**) conversion of activated CAFs to the quiescent state by inhibitors; and (**c**) targeting significant downstream effectors of CAFs. Image created with BioRender.com (accessed on 23 May 2022). CAFs: cancer-associated fibroblasts; TME: tumor microenvironment.

With an increased understanding of CAFs, it is believed that CAFs may serve as promising targets for ovarian cancer therapeutics. Many studies in this review have suggested potential targets as treatment strategies for ovarian cancer, one of which, involving the targeting of TGF-β 1 and 2, is currently under investigation in clinical trials (Phase 2 Trial of Maintenance Vigil for High-Risk Stage IIIb–IV Ovarian Cancer (VITAL), NCT02346747) [107] (Figure 4). In addition, the most common inhibitors targeting CAFs are summarized in Table 2. 

### 5.1. Depletion of CAFs via Specific Surface Markers

Current evidence has indicated that CAFs are important mediators of proliferation, migration, and invasiveness in ovarian cancer. Four CAFs phenotypes in ovarian cancer have been characterized, mainly by the expression levels of α-SMA, FAP, and CD29. Direct CAF-depletion treatment strategies mainly target CAF surface markers, such as α-SMA, FAP, and GPR77 (Figure 4a). In a transgenic mouse model of PDAC, selective depletion of α-SMA could not only restrain angiogenesis but also enhance hypoxia and induce epithelial-to-mesenchymal transition (EMT) and stemness, thus leading to reduced survival [15]. Moreover, knockout of CAFs alters CD4^+^Foxp3^+^ Treg composition to inhibit immune surveillance, thus impeding the response of myofibroblast-depleted tumors to gemcitabine [15]. Despite this, anti-CTLA-4 immunotherapy effectively abrogates tumor development and prolongs survival in mice [15]. However, the clinical outcomes of targeting α-SMA seem to be unsatisfactory; another CAF treatment strategy is to target FAP. Depletion of FAP-expressing CAFs can cause hypoxic necrosis of cancer and stromal cells via interferon-γ (IFN-γ) and tumor necrosis factor-α (TNF-α) [115]. At the same time, FAP-silenced SKOV3 cells can reduce tumor growth and inhibit CAF infiltration [116]. However, FAP is not specifically expressed by CAFs, which may affect the accuracy of CAF-targeted therapies. One study has shown that CD10^+^ GPR77^+^ CAFs can promote successful implantation in xenotransplantation and that targeting GPR77 could inhibit tumor formation and restore tumor chemosensitivity [94]. On the basis of these findings, we hypothesize that depletion of CD10 and/or GPR77 may suppress the tumor-promoting roles of CAFs in ovarian cancer. Further studies are needed to better elucidate the mechanism and function of other specific markers of CAFs. Another strategy to reduce CAFs in tumors is to target potential cellular sources of CAFs. For example, endothelial cells are a potential source of CAFs in malignant pleural mesothelioma (MPeM), and VEGF is a key mitogen in MPeM [113]. Bevacizumab is an antiangiogenic agent against VEGF that can inhibit angiogenesis and tumor growth to reduce CAF infiltration in tumor sites. A phase III clinical study targeting CAF precursors with bevacizumab in MPeM has reported improved overall survival but also observed toxic effects [113]. Bevacizumab is the only approved antiangiogenic agent in ovarian cancer treatment. Several clinical trials have reported that the application of bevacizumab after chemotherapy (carboplatin and paclitaxel) has improved progression-free survival in ovarian cancer [99,114]. CAFs can also secrete small extracellular vesicles (sEVs) which could transport growth factors to target cells. In oral squamous cell carcinoma (SOCC), VEGF released from CAFs could bind to sEVs and stimulate angiogenesis and impair cell sensitivity to bevacizumab [117]. Furthermore, heparinase could release VEGF from sEVs and bevacizumab could neutralize the dissociated VEGF and inhibit angiogenesis [117].

### 5.2. Conversion of Activated CAFs to the Quiescent State

Besides direct depletion of CAFs, another therapeutic strategy is to revert activated CAFs to the quiescent state or induce their functioning as tumor-suppressive players (Figure 4b). A number of cytokines or pathways are involved in the CAFs activation process. For example, microRNAs reprogram normal fibroblasts into cancer-associated fibroblasts in ovarian cancer, especially by downregulation of miR-31 and miR-214 and upregulation of miR-155. CCL5, a direct candidate target of miR214, can contribute to suppressing tumor growth and metastasis [38]. Ovarian cancer-related fibroblasts have been shown to exhibit resistance to PARP inhibitors (PARPis). At the same time, the activation of PARPi-induced stromal fibroblasts requires increased CCL5 secretion by the activation of NF-κB signaling [118]. Therefore, neutralizing CCL5 may facilitate the transition of CAFs from an activated state to a quiescent state. In addition, TGF-β may contribute to the activation of CAFs and the progression of ovarian cancer. Two autocrine loops, mediated by TGF-β and SDF-1, are stimulated to initiate and maintain the differentiation of human mammary fibroblasts into activated CAFs, which have tumor-promoting features [37]. Targeting TGF-β could weaken both the expression of CXCR4 and activation of TβR–Smad signaling in stromal fibroblasts, thus hindering the feedback loop of TGF-β and SDF-1 and blocking the activation of tumor-promoting CAFs [37]. The targeting of other related genes, such as *MYC* and *VCAN*, may serve as potential therapeutic targets for ovarian cancer [48,79]. Indeed, treatment with vitamin D in PDAC has shown a 57% increase in survival compared to chemotherapy alone in mice [119]. Application of vitamin A has also been demonstrated to improve survival in PDAC [120]. Therefore, we hypothesize that vitamins can be used as adjuncts to current ovarian cancer treatments to enhance their therapeutic efficacy.

### 5.3. Targeting Significant Downstream Effectors of CAFs

Challenges remain in the development of strategies for CAF depletion or differentiation into a quiescent state. Therefore, alternative strategies have been developed to target other important effectors involved in CAFs biological processes (Figure 4c). Studies have shown that TGF-β plays multifaceted roles in defining tumor-immune phenotypes. CAFs are more sensitive to immune checkpoint inhibitors (ICIs) in immune-infiltrated tumors due to higher antigen presentation, PD-L1 expression, and immune features [121]. TGF-β is correlated with weak responses to ICI therapy in immune-desert and immune-excluded tumors [19]. Coadministration of TGF-β inhibitors and anti-PD-L1 antibodies can exhibit anti-tumor immunity by facilitating T cell infiltration in tumor sites [109]. TGF-β is highly expressed in ovarian cancer and is essential for activating CAFs, facilitating tumor pathogenesis processes, averting immune regulation, and eventually forming a favorable TME. A clinical trial of an autologous tumor vaccine named gemogenovatucel-T (Vigil) (NCT02346747) has shown that Vigil provided personal neoantigens, downregulated TGF-β 1 and 2 and induced GMCSF expression, leading to systemic immunosuppressive activities. Current results have indicated long-term beneficial effects of Vigil with respect to primary endpoints and support further investigation of Vigil in ovarian cancer [110]. Studies have found that inhibiting the CXCR4/CXCL12 axis can increase sensitivity to anti-PD-1 and anti-CTLA4 immunological therapies [41,56]. IL-6 is specially induced by IL-1β, TNF-α, and TGF-β signaling. Elevated IL-6 levels in serum and ascites have been shown to be associated with impaired cancer cell sensitivity to chemotherapy and poor prognosis in ovarian cancer patients. IL-6 can induce the overexpression of MMP, promote EMT, and activate the MAPK, PI3K/AKT, or JAK/STAT3 pathways to enhance tumor growth and cause chemoresistance [83]. Tocilizumab, an IL-6R antibody, could induce the production of tumor immunity-stimulating factors, such as IL-12, IL-1β, and anti-tumor cytokines, such as TNF-α, and IFN-γ. Clinical trial results have revealed that the combination of Tocilizumab with chemotherapy showed a good safety profile and possibly restored cancer cell sensitivities to chemotherapeutic agents and achieved immunological benefits [84,108]. However, there are certain limitations to these studies, such as small numbers of enrolled patients, serum levels detected at different FIGO stages, and no parallel analysis of IL-6 with other cytokines [84]. Therefore, further studies are needed to clarify the roles of IL-6 and IL-6R in ovarian cancer pathogenesis and treatment responses. It is reported that PS1 is highly expressed in CAFs and plays an important role in regulating effector CD8+ T cells in ovarian cancer. Notably, when PS-1 expression is silenced, immunosuppressive IL-1β is downregulated via the WNT/β-catenin pathway and consequently contributes to the proliferation of functional CTLs to improve the efficacy of immunotherapies [55]. Another important effector associated with CAF functions is the immunosuppressive enzyme CD73 on the surface of CAFs. In vitro, CD73 and extracellular adenosine can promote tumor growth and induce the expression of the BCL-2 anti-apoptotic family [122]. There are studies that have applied Hedgehog inhibitors in attempts to restrain tumor growth in gastrointestinal cancer. One study has shown that Hedgehog signaling inhibitors are associated with alterations of fibroblast composition and effector T cell infiltration and that the altered microenvironment facilitates pancreatic cancer progression [123,124]. Meanwhile, autophagy plays an important role in regulating the metabolic crosstalk between ovarian cancer cells and cancer-associated fibroblasts. In this respect, therapeutic options are referred to both induction and inhibition of autophagy. Compared to monotherapy, combined administration of PARPi and EGFR inhibitors has shown better antitumor effects in ovarian cancer A2780 xenografts, mainly through the activation of the ERK (MAPK) and JNK and inhibition of the AKT/mTOR/(p70S6K) pathways [111]. Interestingly, suppression of autophagy is partly associated with the improved efficacy of cisplatin in cisplatin-resistant ovarian cancer cells. For example, the autophagy inhibitor elaiophylin can exhibit anti-tumor activities as a single agent and can also decrease cell viability in combination with cisplatin. Therefore, elaiophylin has the potential to be a treatment agent for ovarian cancer [112].

Compared with monotherapy, combinations of chemotherapy, immunotherapy, and/or radiotherapy may achieve better therapeutic efficacy. Studies have shown that fibroblasts inhibit platinum aggregation in tumor nuclei by producing cysteine and glutathione, while CD8+ T cells can produce γ-interferon to upregulate γ-glutamyltransferase and inhibit the transcription of xc (−) cystine and glutamate receptors through the JAK/STAT1 pathway, thus controlling the synthesis of glutathione and cysteine in fibroblasts and effectively eliminating chemotherapy resistance in matrices [92]. Recent clinical trial results have reported that the application of bevacizumab after chemotherapy has been associated with survival benefits in ovarian cancer patients. Benefits have also been observed when bevacizumab has been used in combination with PARPis [99,114]. Antiangiogenics can enhance cancer immunotherapy by increasing immune cell infiltration and reducing immunosuppression in the TME [99]. In immune-desert tumors, low-dose radiotherapy combined with immunotherapy could reprogram the microenvironment and mobilize both innate and adaptive cells to achieve NKG2D-dependent anti-tumor immunity [101].

## 6. Conclusions

Fulfilling important roles in the TME, CAFs have attracted wide attention and great efforts have been made in understanding their phenotypes, features, and heterogeneity. The current review first described the potential cellular sources and activators of CAFs, as well as several functionally distinct subtypes of CAFs. We next summarized the various roles of CAFs in regulating tumor pathogenesis and drug resistance. An increasing number of CAF-targeted therapies are under investigation in clinical or pre-clinical studies. 

Studies utilizing novel biological technologies and optimized animal models have indicated that CAFs may be promising therapeutic targets. It is important to better understand the crosstalk of CAFs with cancer cells, immune cells, and other components in the TME. It is possible that CAFs subtypes and distinct markers could be helpful in developing promising therapeutic strategies. To date, few clinical trials have directly focused on the effects of anti-angiogenic agents or other monoclonal antibodies in CAFs, and limited clinical success targeting CAFs has been achieved. Well-designed clinical studies with adequate sample sizes are needed to provide evidence and achieve the transformation of the basic research on CAFs into clinical practice and finally obtain clinical benefits. Future studies should also focus on clarifying the explicit roles of specific CAFs subtypes in interplay with different molecules and the exact mechanisms of therapeutic resistance. Detailed knowledge about CAFs and specific mechanisms will help design or refine potential and prospective modalities for ovarian cancer treatment.

## Figures and Tables

**Table 1:** Mechanisms used by CAFs to promote ovarian cancer biological processes.

Perspectives	Mediators	Mechanisms	Reference
Facilitate tumor growth, proliferation, and metastasis	CTHRC1	Activates EGFR/ERK1/2/AKT signaling pathway	[45]
	COL11A1	Activates ERK pathway; induces activation of TGF-β1-MMP3 axis	[46,47]
	VCAN	Activates NF-κB pathway; upregulates expression of CD44, MMP-9, and the hyaluronan-mediated motility receptor	[48]
	HGF	Activates the c-Met/PI3K/Akt and GRP78 signaling pathways	[49]
	FGF-1	Activates FGF-1/FGF-4 signaling; activates the MAPK/ERK pathway; upregulates EMT-related genes	[50]
Avert immune regulation	TGF-β	Inhibits effector immune cells; regulates innate and adaptive immune cells	[51,52,53,54]
	PS-1	via the WNT/β-catenin pathway	[55]
	CXCL12	CXCR4-dependent pathway; prevents effector T cells from making contact with cancer cells; enhances recruitment of Tregs	[41,56]
	IL-33	Modifies the immune microenvironment; drives type 2 immunity	[57]
	IL-6	Activates the STAT3 pathway; induces transformation of CD8+ T cells into highly cytotoxic cells	[58]
Metabolic regulation	p38αMAPK	Provides a tunnel for glycolysis	[59]
	CXCL14	Upregulates the expression of long non-coding RNALINC00092; alters glycolysis	[60]
	ROS	Induces autophagy, mitophagy, and glycolysis via NF-κB activation, with upregulation of MCT4 and loss of Cav-1 expression	[61,62]

AKT: protein kinase; Cav-1: caveolin-1; CTHRC1: collagen triple helix repeat containing-1; COL11A1: collagen type XI alpha 1 chain; CXCL12: C-X-C chemokine ligand 12; CXCL14: C-X-C chemokine ligand 14; EGFR: epidermal growth factor receptor; ERK: extracellular signal-regulated kinase; EMT: epithelial–mesenchymal transition; FGF-1: fibroblast growth factor-1; GRP78: glucose-regulating protein 78; HGF: hepatocyte growth factor; IL-6: interleukin-6; IL-33: interleukin-33; MAPK: mitogen-activated protein kinases; MMP3: matrix metalloproteinase-3; MCT4: monocarboxylate transporters 4; PFKFB2: 6-phosphofructo-2-kinase/fructose-2,6-biphosphatase 2; PI3K: phosphatidylinositol 3-kinase; PS-1: presenilin-1; STAT3: signal transducer and activator of transcription 3; TGF-β: transforming growth factor-beta; VCAN: versican.

**Table 2:** CAFs-associated inhibitors in ovarian cancer.

Inhibitors	Targets	Functions	Reference
Tocilizumab	IL-6R	Promotes anti-tumor immunity	[108]
INCB28060	HGF and c-Met	Blocks chemotherapeutic failure	[49]
PD173074	FGF	Terminates cellular proliferation and migration	[50]
Galunisertib	TGF-β1	Reverses MHC-I loss caused by TGF-β	[72]
DNMT	MHC-I	Inhibits tumor-immune excluded subtype	[52]
MPDL3280A	PD-L1	Suppresses T cell migration, proliferation, and secretion of cytotoxic mediators and restricts tumor cell killing	[109]
Vigil	TGFβ-1 and TGFβ-2	Downregulates TGF-β 1 and 2; provides personal neoantigen; induces GMCSF expression	[110]
AG1478	EGFR	Upregulates autophagy levels	[111]
Elaiophylin	Autophagy	Inhibits activation of autophagy	[112]
Bevacizumab	VEGF	Inhibits tumor angiogenesis	[99,113,114]

CAFs: cancer-associated fibroblasts.

## Abbreviations

CAFs	cancer-associated fibroblasts
CAV1	caveolin1
CD29	integrin β1
COL11A1	collagen type XI alpha 1 chain
CXCR4	C-X-C chemokine receptor type 4
CXCL12	C-X-C chemokine ligand 12
CXCL14	C-X-C chemokine ligand 14
ECM	extracellular matrix remodeling
EMT	epithelial-mesenchymal transition
ERK	extracellular signal-regulated kinase
FAK	focal adhesion kinase
FAP	fibroblast activation protein
FGF-1	fibroblast growth factor-1
GRP78	glucose-regulating protein 78
HGF	hepatocyte growth factor
GPR77	G protein-coupled receptor 77
MAPK	mitogen-activated protein kinase
MCT4	monocarboxylate transporters 4
MDSCs	myeloid-derived suppressor cells
MMP3	matrix metalloproteinase-3
MSCs	mesenchymal stem cells
PARP	poly ADP-ribose polymerase
PDAC	pancreatic ductal adenocarcinoma
PDGF	platelet-derived growth factor
PI3K	phosphatidylinositol 3-kinase
PDGFRα/β	platelet-derived growth factor receptor-α/-β
PFKFB2	6-phosphofructo-2-kinase/fructose-2,6-biphosphatase 2
PS-1	presenilin-1
p38αMAPK	p38alpha mitogen-activated protein kinase
STAT3	signal transducer and activator of transcription 3
TGF-β	transforming growth factor-beta
TIME	tumor immune microenvironment
TME	tumor microenvironment
VCAN	versican
α-SMA	α-smooth muscle actin
